# Multi-antigen-targeted chimeric antigen receptor T cells for cancer therapy

**DOI:** 10.1186/s13045-019-0813-7

**Published:** 2019-11-29

**Authors:** Xiao Han, Yao Wang, Jianshu Wei, Weidong Han

**Affiliations:** Molecular and Immunological Department, Bio-therapeutic Department Chinese PLA General Hospital, No. 28 Fuxing Road, Beijing, 100853 China

**Keywords:** Chimeric antigen receptor T cells, Logic gate, Antigen escape, On-target, Off-tumor toxicities

## Abstract

The approval of two chimeric antigen receptor-modified T cell types by the US Food and Drug Administration (FDA) for the treatment of hematologic malignancies is a milestone in immunotherapy; however, the application of CAR-T cells has been limited by antigen escape and on-target, off-tumor toxicities. Therefore, it may be a potentially effective strategy to select appropriate targets and to combine multi-antigen-targeted CAR-T cells with “OR”, “AND” and “NOT” Boolean logic gates. We summarize the current limitations of CAR-T cells as well as the efficacy and safety of logic-gated CAR-T cells in antitumor therapy. This review will help to explore more optimized strategies to expand the CAR-T cell therapeutic window.

## Introduction

Chimeric antigen receptor (CAR)-T cell therapy has shown dramatic results for hematological malignancies. Recently, two types of CD19-targeted CAR-T cells, tisagenlecleucel (Kymriah), and axicabtagene ciloleucel (Yescarta) were approved by the FDA for the treatment of relapsed/refractory (R/R) B-cell acute lymphoblastic leukemia (B-ALL) and certain types of large B cell lymphoma [1, 2].

CAR-T cell therapy represents a landmark in the field of cancer immunotherapy along with immune checkpoint inhibitors, including programmed death-1 (PD-1) and cytotoxic T lymphocyte-associated protein-4 (CTLA-4) blockade. Patients with a high mutation burden have an ideal clinical response to immune checkpoint inhibitors, whereas patients with low mutation burdens are less responsive [3]. Encouragingly, CAR-T cells have been confirmed to have high efficacy for patients with a low mutation burden [4].

One key to the clinical success of CAR-T therapy is the selection of an ideal target, which should be uniformly expressed on the surface of tumor cells and should not be expressed on vital healthy cells. Despite inducing B cell aplasia, which is clinically manageable, CD19 is considered a promising target based on its expression on B cell malignancies [5]. However, multi-center clinical trials have demonstrated that the relapse rate of CD19-targeted CAR-T cell therapy is approximately 30% [6], which may be associated with the lack of CAR-T cell persistence and antigen escape, including antigen downregulation or even antigen loss [7]. As an important mechanism for evading CAR-T cell therapy, antigen escape also occurs in non-B cell malignancies as well as in solid tumors [8, 9]. Another important factor limiting CAR-T therapy is on-target, off-tumor toxicities. The targeting of CD19+ has been shown as a viable strategy, whereas the on-target, off-tumor toxicities from other targeted antigens may be unacceptable or even fatal.

Therefore, the combination of multi-antigen targeting may be a potential strategy to expand the effectiveness of this novel immunotherapy. The Boolean logic gates of “AND”, “OR”, and “NOT” have been applied to manipulate multi-antigen targeted CAR-T cells to prevent tumor antigen escape and to alleviate on-target, off-tumor toxicities. In this review, we will summarize the limitations of the current clinical applications of CAR-T cell therapy, mainly focusing on the mechanism of antigen escape that leads to disease relapse and CAR-T cell-related on-target, off-tumor toxicities. In addition, we will explore rational strategies to overcome these barriers, thereby increasing the safety and efficacy of CAR-T cells.

## Limitations of CAR-T cell therapy in clinical application

### Limitation 1: Disease relapse

There are two main forms of disease relapse after CAR-T cell infusions: antigen-positive relapse in the early phase and antigen escape relapse in a later phase. The disease relapse of antigen-positive cells is closely related to CAR-T cell persistence. Factors that affect the persistence of CAR-T cells require further exploration, of which the T cell-intrinsic quality and CAR structure are critical. Substituting fully humanized single-chain variable fragments (scFvs) for murine scFvs can reduce immune-mediated resistance, thus improving the persistence of CAR-T cells [10]. In the CAR structure, the co-stimulatory signal domain also has an important effect on CAR-T cell therapy. Preclinical and clinical trials have demonstrated that the 4-1BB (CD137) costimulatory domain induced the longer-term persistence of CAR-T cells than CD28 [11, 12]. Tonic signaling induced by CAR clustering promotes CAR-T cell exhaustion but CD137 costimulatory domain might potentially ameliorate this effect [13]. The CD137, which is significantly different from the CD28 costimulatory domain, has been shown to stimulate fatty-acid oxidation in metabolism and to increase the central memory phenotype, thereby improving CAR-T cell persistence [14].

Antigen escape, including antigen loss or downregulation, is an important mechanism driving disease relapse following CAR-T cell immunotherapy. Alternative splicing is the primary cause of CD19 antigen loss and induces the generation of CD19 isoforms that gradually develop into dominant clusters under the strong immune pressure of adoptive T cell therapy (ACT), thereby escaping CD19-targeted CAR-T cell–mediated cell death [15, 16]. Complete antigen loss is not necessary for CAR-T cell resistance, and in some cases, a reduction in antigen density is sufficient to evade CAR-T cells [17]. Nevertheless, a minimum antigen density threshold is required for CAR-T cells to achieve therapeutic efficacy. In preclinical models, Sadelain et al. demonstrated that CAR-T cells extract and transfer target antigens into T cells via trogocytosis, which leads to a reversible decrease in antigen density. In addition, researchers also verified that combinatorial multi-antigen-targeted strategies could effectively prevent the tumor escape caused by low antigen density that results from CAR-T cell trogocytosis [18].

### Limitation 2: On-target, off-tumor toxicities

Most of the selected antigens are not tumor-specific but are only overexpressed on tumor cells, making it difficult for CAR-T cells to kill targeted tumor cells in a safe manner. B lymphocyte aplasia is a clinically manageable on-target, off-tumor toxicity that is prevalent in the treatment of hematological malignancies with CD19-targeted CAR-T cells [19]. However, during solid tumor treatment, on-target, off-tumor toxicities following the infusion of CAR-T cells can occasionally pose serious or even fatal threats [20]. An early clinical trial revealed an unexpected grade 2 to 4 hepatotoxicity after several infusions of genetically engineered T cells retargeting carbonic anhydrase IX (CAIX) in the treatment of metastatic renal cell carcinoma. Liver biopsies showed that T lymphocytes infiltrated around the bile duct, and there were CAIX-positive bile duct epithelial cells. These observations are sufficient to demonstrate that CAIX-targeted CAR-T cells attack CAIX+ bile duct epithelial cells, resulting in hepatotoxicity [21]. Although target antigens are expressed on both tumor and normal cells, not all kinds of CAR-T cell therapies exhibit observable on-target, off-tumor toxicities, such as targeting carcinoembryonic antigen (CEA), mesothelin (MSLN), and Interleukin 13 receptor, Alpha2 (IL13Rα2) [22–24]. Furthermore, CAR-T cells targeting the same antigen have demonstrated different on-target, off-tumor toxicities in preclinical trials, some of which were mild, whereas some were lethal, such as targeting fibroblast activation protein-α (FAP) [25–27].

Affinity is another important factor that affects the activity of CAR-T cells. High affinity can cause CAR-T cells to recognize target antigens even at a low density and to proliferate and kill tumor cells, but it can also increase the severity of off-tumor toxicities. However, when the affinity exceeds a certain threshold (*K*_d_ < 10^−8^ M) it makes no significant difference in regard to promoting the efficiency of T cells [28]. Spencer Park et al. demonstrated that micromolar affinity CAR-T cells had stronger antitumor activity and lower off-tumor toxicities than their nanomolar affinity counterparts [29]. Therefore, exploring a rational strategy for regulating CAR-T cell affinity is necessary for controlling the on-target, off-tumor toxicities. It is important to balance the relationship between potency and toxicity in CAR-T cell therapy; therefore, June et al. applied the concept of the “therapeutic window”, derived from drug toxicology, to the field of CAR-T cell therapy to achieve the highest therapeutic benefit with acceptable toxicity, thereby expanding and optimizing the clinical application of CAR-T cells in solid tumors [30].

Notably, genetically engineered T cells, which are mediated by lentiviral vectors or zinc-finger nuclease (ZFN), similar to other genetic engineering therapies, have the potential risks of insertional mutagenesis causing on-tumor, off-target toxicities [31]. In addition, genome-editing techniques have been applied to knock out T cell receptor (TCR) complexes, causing endogenous TCR deficiency in T lymphocytes and avoiding unpredictable off-tumor toxicities [32].

## Strategies combining multi-antigen-targeted CAR-T cells

**Fig. 1:**
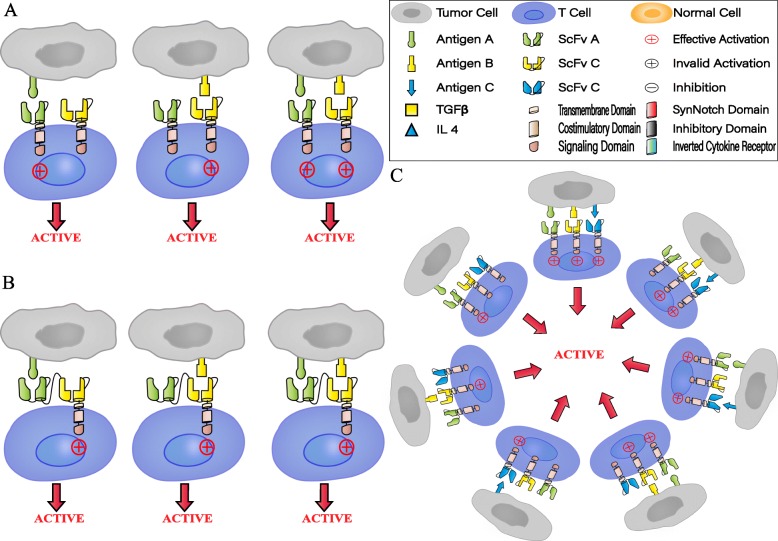
“OR” logic-gated CAR-T cells for preventing tumor antigen escape. **a** Dual CAR-T cells: “OR” logic gate. Each CAR contains a complete signal domain that activates the antitumor effect of CAR-T cells in the presence of either cognate antigen. **b** Tandem CAR-T cells: “OR” logic gate. One CAR coexpresses two distinct antigen-binding domains in tandem, using the “OR” logic gate to activate T cell. **c** Trivalent CAR T cells: “OR” logic gate. Three CARs in one T cell utilize the “OR” logic gate to kill tumor cells in the presence of either validated antigen

**Fig. 2:**
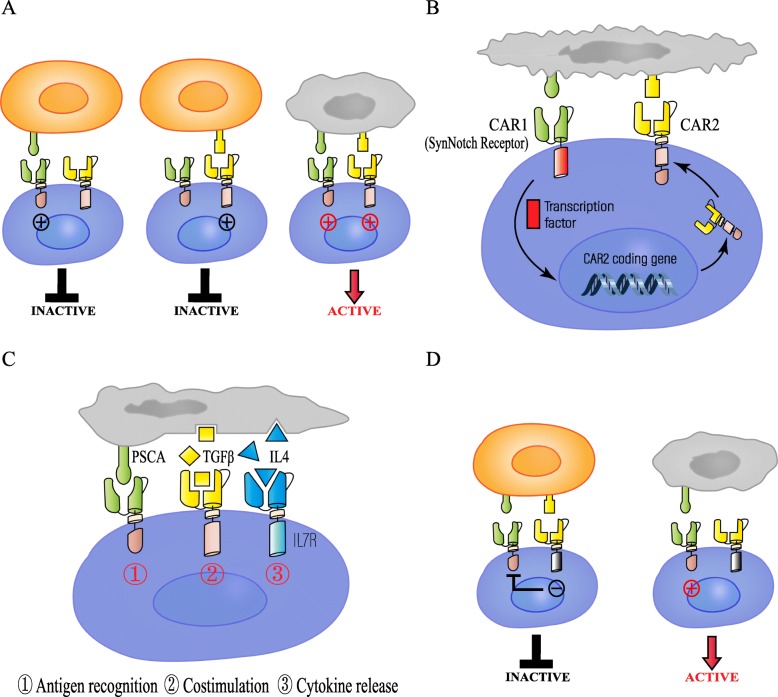
“AND” and “NOT” logic-gated CAR-T cells for alleviating on-target, off-tumor toxicities. **a** Dual CAR-T cells: “AND” logic gate. Two distinct CARs are coexpressed with complementary signaling domains in one T cell that fully activates the T cell only in the presence of both cognate antigens. **b** Synthetic Notch Receptor System: “AND” logic gate. In the presence of cognate antigen of CAR1, SynNotch receptor induces the conditional expression of CAR2 in a transcriptional manner, thereby targeting to the second antigen, and finally achieving highly specific activation of T cell. **c** Trivalent CAR T cells: “AND” logic gate. Trivalent CAR-T cell response to tumor-specific expression patterns to overcome the immunosuppression of TME, rather than adding additional CARs targeting TAAs. **d** Dual CAR-T cells: “NOT” logic gate. iCAR-T cells selectively kill target cells only expressing one antigen, whereas off-target cells co-expressing another inhibitory ligand recognized by iCAR were protected from attack, allowing T cells to distinguish target cells from the off-target cells

As antigen escape is the main potential mechanism for evading immunotherapy, the combination of multi-antigen-targeted CAR-T cells is being tested as a strategy to reduce relapse [33]. Several major multi-antigen-targeted CAR-T cell therapies at present include the following: (I) Pooled CAR-T cells: mixtures of two engineered T cell lines, each of which expresses a distinct antigen-specific CAR; (II) Dual CAR-T cells: two individual CARs that target different antigens in a single T cell; (III) Tandem CAR-T cells: two distinct antigen-binding domains are connected in tandem to a single CAR; and (IV) Trivalent CAR T-cells: three CARs target specific antigen molecules in a single engineered T cell. Based on the multi-antigen-targeting strategy, Boolean logic has been applied to “gate” the activity of CAR-T cells to achieve high efficiency and low toxicity. For instance, T cells with two independent CAR molecules or a pool mixed with different specific CAR-T cells can use the “OR” logic gate, which enables CAR-T cells to antitumor in the presence of either targeted antigen. However, the “AND” logic-gated CAR-T cells can only be activated in the presence of both antigens simultaneously. In addition, “NOT” logic gate can also be used to make engineered T cells distinguish target cells from non-target cells, avoiding attacks on normal tissue, thereby achieving the safety of CAR-T cells (Table 1) (Fig. 1, 2).

**Table 1:** Strategies combining multi-antigen-targeted CAR-T cells

Boolean logic gates	CAR-T cell mode	Target	CAR construction	Indication	Reference
OR	Pooled CAR-T cell	EGFR and CD133	EGFR-CD137-CD3ζ CD133-CD137-CD3ζ	CAA	Feng et al. 2017
	Dual CAR-T cell	HER2 and IL-13Rα2	HER2-CD28-CD3ζ IL13Rα2-CD28-CD3ζ	GBM	Hegde et al. 2013
		CD19 and CD123	CD19-CD137-CD3ζ CD123-CD137-CD3ζ	B-ALL	Ruella et al. 2016
	Tandem CAR-T cell	CD19 and HER2	CD19-HER2-CD28-CD3ζ	Experimental tumors	Grada et al. 2013
		HER2 and IL13Rα2	HER2-IL13Rα2-CD28-CD3ζ	GBM	Hegde et al. 2016
		CD19 and CD20	CD19-CD20-CD28- CD137-CD3ζ	B cell malignancies	Zah et al. 2016
		CD20 and HER2	Nano(CD20-HER2)-CD28-CD3ζ	Experimental tumors	De Munter et al. 2018
		CD22 and CD19	CD22-CD19-CD137-CD3ζ	R/R B-ALL	Jia et al. 2019
	Trivalent CAR T cell	HER2, IL13Rα2 and EphA2	HER2-CD28-CD3ζ IL13Rα2-CD28-CD3ζ EphA2-CD28-CD3ζ	GBM	Bielamowicz et al. 2018
AND	Dual CAR-T cell	MSLN and FRa	MSLN-CD3ζ FRa-CD28	Ovarian cancer	Lanitis et al. 2013
		CD19 and MSLN GFP and CD19	α-CD19 SynNotch/α-MSLN-CD137-CD3ζ α-GFP SynNotch/α-CD19-CD137-CD3ζ	Experimental tumors	Roybal et al. 2016
	Trivalent CAR T cell	PSCA, TGFβ and IL4	PSCA-CD28-CD3ζ TGFβ-CD137 IL4Rα-IL7Rα	Pancreatic cancer	Sukumaran et al. 2018
NOT	Dual CAR-T cell	PSMA and CD19	PSMA-PD1/CTLA-4 CD19-CD28-CD3ζ	Experimental tumors	Fedorov et al. 2013

*EGFR* epidermal growth factor receptor; *CAA* cholangiocarcinoma; *HER2* Human epidermal growth factor receptor-2; *IL13Rα2* Interleukin 13 Receptor, Alpha2; *GBM* Glioblastoma; *R/R* Relapsed and refractory; *B-ALL* B cell acute lymphocytic leukemia; *MSLN* mesothelin; *FRa* A-folate receptor; *GFP* Green fluorescent protein; *TGFβ* Transforming growth factor β; *IL4* Interleukin 4; *PSCA* Prostate stem cell antigen; *PSMA* prostate-specific membrane antigen; *PD-1* Programmed death-1; *CTLA-4* Cytotoxic T lymphocyte-associated protein-4

### “OR” logic–gated CAR-T cells for preventing tumor antigen escape

#### Pooled CAR-T cells using the “OR” logic gate

Pooled CAR-T cells are a mixture of two CAR-T cell lines, each targeting different cognate antigens, thereby achieving lower tumor relapse through an “OR” logic gate. This strategy has been investigated, such as pooling monospecific CAR-T cells targeting human epidermal growth factor receptor-2 (HER2)/IL-13Rα2 for glioblastoma and CD19/CD123 for B-ALL [34, 35]. In terms of cytokine secretion and cytolysis, pooled CAR-T cells exhibited lower levels than tandem CAR-T cells and dual CAR-T cells but higher levels than the individual CAR-T cell lines. It is worth noting that the use of two CAR-T cell lines places strong immune pressure on the tumor cells, which may lead to the simultaneous escape of both antigens. In addition, the simultaneous introduction of two CAR-T cell lines may lead to an imbalance in the cell population. The significant amplification of CD19-targeted CAR-T cells, which was higher than the amplification of CD20-targeted CAR-T cells, was observed during co-infusion despite the persistence of the CD20 antigen [36].

In addition to the simultaneous mixture of two CAR-T cell lines, a method termed cocktail immunotherapy, which involves the sequential administration of different antigen-targeted CAR-T cells, has also been used in clinical trials. Our team reported a case of a female patient with advanced unresectable/metastatic cholangiocarcinoma (CAA) who was resistant to both radiotherapy and chemotherapy. We successfully infused this patient with epidermal growth factor receptor (EGFR)- and CD133-targeted CAR-T cells separately.

The patient underwent two cycles of EGFR-targeted CAR-T cells infusion and achieved 8.5-month of partial remission (PR) until tumor progression was detected by positron emission tomography-computed tomography (PET-CT). Thus, another cycle of EGFR-targeted CAR-T cells combined with anti-PD-1 monoclonal antibody was administration. Subsequent PET-CT revealed newly emerged metastases and previous abdominal tumor enlargement. Since most tumor cells expressed CD133, the patient was enrolled in the clinical trial of CD133-targeted CAR-T cell. Radiographic evaluation of metastatic tumors showed a significant reduction or even disappearance with the CD133-targeted CAR-T cell administration, and the patient obtained 4.5-month PR. It is worth noting that both batches of CAR-T cells have caused acute adverse reactions associated with the infusion, among which CD133-targeted CAR-T cell-related acute subcutaneous hemorrhage is serious, requiring clinical emergency intervention [37].

#### Dual CAR-T cells using the “OR” logic gate

Dual CAR-T cells are individual T cells that are engineered to co-express two separate CARs specific for cognate antigens with two individual signals. Depending on the signal transmission pattern, Dual CAR-T cells have various logic-gate operations, including “OR”, “AND”, and “NOT”.

Due to tumor heterogeneity, there are no single specific tumor antigens. Under the strong immune pressure that occurs during immunotherapy, the tumor target antigens tend to selectively escape. This phenomenon is also present in CAR-T cell therapy for glioblastoma (GBM). The combinational multi-antigen targeted strategy may offset the potential antigen escape mechanism. Therefore, Meenakshi et al. generated “OR” logic-gated CAR-T cells that contained distinct CAR molecules targeting two glioma-restricted antigens including HER2 and IL13Rα2 (HER2-targeted scFv CD28ζ and IL13Rα2- targeted scFv-CD28ζ) [34]. Compared with the pooled populations of HER2-targeted and IL13Rα2-targeted CAR-T cells and monospecific CAR-T cells, the HER2- “OR” IL13Rα2-targeted CAR-T cells more effectively prevented antigen escape and enhanced antitumor efficacy.

This enhancement of antitumor activity may have resulted from the increased ζ-chain signaling by the simultaneous binding of HER2 and IL13Rα2. When dual CAR-T cells were exposed to a single HER2 or IL13Rα2 antigen, the signal transduction intensity was equivalent to that of natural T cells, whereas when dual CAR-T encountered the co-presence of both antigens, maximal downstream signal transduction was exhibited, which was verified by the detection of high levels of zeta-chain-associated protein kinase 70 (ZAP-70) phosphorylation. In addition, studies have confirmed that the density balance between tumor target antigens, and CAR molecules has an important influence on the T cell response. Dual CAR-T cells are advantageous because they have a high-density of CAR molecules on the surface of the T cells and target tumor cells with a high density of cognate antigens [34].

CD123 was shown to be highly expressed in most patients with primary B-ALL, especially in patients with CD19-negative relapse following the administration of CD19-targeted CAR-T cells [37]. Based on this phenomenon, effective strategies against antigen escape relapse were devised. Marco Ruella et al. generated CD19- “OR” CD123-targeted CAR-T cells with two complete second-generation CAR structures that included CD19-targeted scFv CD137ζ and CD123-targeted scFv CD137ζ. Compared with monospecific CAR-T cells and pooled CAR-T cells, Dual CAR-T cells exhibited superior antitumor efficacy and longer-term persistence. Interestingly, based on confocal imaging, the two CARs of dual CAR-T cells were involved in the establishment of the same immune synapse with lymphoma cells [35] (Fig. 1a)

#### Tandem CAR-T cells using the “OR” logic gate

Tandem CAR-T cells are generated by modifying individual T cells to co-express distinct scFvs in tandem using the “OR” logic gate. Preclinical trials have provided a proof-of-concept for Tandem CAR-T cells, such as cells simultaneously targeting CD19 and HER2 (CD19/HER2-targeted scFv- CD28/CD3ζ), HER2 and IL13Rα2 (HER2/IL13Rα2-targeted scFv-CD28/CD3ζ) and CD19 and CD20 (CD19/CD20-targeted scFv-CD28/CD137/CD3ζ) [36, 38, 39]. Tandem CAR-T cells caused a distinct response to each of the two cognate antigens, preserving the cytolytic capacity when one of the cognate antigens escaped. In addition, synergistic antitumor efficacy has been demonstrated upon simultaneously encountering both antigens. The formation of bivalent immune synapses in the process of tandem CAR-T cell exposure to tumors co-expressing both cognate antigens is crucial for T cells to effectively prevent antigen escape and to improve antitumor efficacy [39].

However, some factors have hampered the preparation and application of tandem CAR-T cells, such as the cross-pairing of the variable heavy (VH) and variable light (VL) chains between different scFvs, the limitations of viral vector package sizes, and the immunogenicity of scFvs derived from mice [40]. Fortunately, nanobodies are potential substitutes for scFvs because of their structural specificity, including the absence of light chains, very small structures, mutual noninterference, and weak immunogenicity [41]. De Munter et al. generated and verified nano-based tandem CAR-T cells targeting CD20 and HER2 [42].

Our group reported a clinical case of R/R B-ALL in an adult patient. With the administration of Tandem CAR-T cells co-targeting CD22 and CD19 (CD22/CD19-targeted scFv-CD137/CD3ζ) after hematopoietic stem cell transplantation (HSCT), more than 14 months of minimal residual disease (MRD)-negative complete remission (CR) was achieved; the patient developed graft-versus-host disease (GVHD), which is clinically manageable. The treatment process of this 22-year-old patient was relatively complicated. He had received a CD19-targeted CAR-T cell protocol a month prior to the tandem CAR-T cell protocol, in which CAR-T cells were administered following cytoreduction and lymphodepleting chemotherapy. Although this is a single clinical case, tandem CAR-T cells have exhibited superior persistent remission in the treatment of R/R B-ALL [43] (Fig. 1b).

#### Trivalent CAR T cells using the “OR” logic gate

A trivalent CAR T-cell is a single engineered T cell with three CARs targeting validated antigens. As shown by the data from the Meenakshi group above, in response to the complexity of the GBM microenvironment, the high heterogeneity of tumor cells and the low permeability of the blood-brain barrier, dual CAR-T cells co-targeting glioma-restricted antigens exhibited significant advantages in regard to inhibiting antigen escape and enhancing T cell activation and persistence [34]. However, due to the specific antigen pair variation between GBM patients, bispecific CAR-T cells still provide insufficient antigen coverage. Recently, trivalent peptide vaccines targeting glioma-restricted antigens, including EphA2, IL13rα2, and survivin, have been proven to be safe and feasible for the treatment of pediatric GBM [44]. Notably, CAR-T cell therapy can be unrestricted by the human leukocyte antigen (HLA), whereas the efficacy of vaccines has been hampered by the HLA. Therefore, to explore a more universal ACT for GBM patients, the Meenakshi group designed and validated trivalent CAR-T cells that simultaneously targeted HER2, IL13Rα2, and EphA2, which achieved nearly 100% clearance of GBM tumor cells. This powerful antitumor effect of trivalent CAR-T cells in preclinical experiments is potentially due to enhanced transmission signal activation, expanded tumor antigen coverage and robust immune synapse formation [45] (Fig. 1c).

Despite broadening CAR-T cell applications, multi-antigen-targeted strategies also carry risks associated with specific reduction and on-target, off-tumor toxicities. For these adverse reactions with the “OR” gated logic, the CAR-T cells modified with “AND” and “NOT” logic gate can be an optimal approach.

### “AND” and “NOT” logic-gated CAR-T cells for alleviating on-target, off-tumor toxicities

#### Dual CAR-T cells using the “AND” logic gate

With the introduction of a costimulation signal domain into the CAR-T cell structure, second-generation CAR-T cells exhibited long-term persistence and enhanced antitumor efficacy; however, they caused worse on-target, off-tumor toxicities to normal tissues expressing low levels of the tumor-associated antigen (TAA). To address this problem, Lanitis et al. proposed a dual-signaling strategy that physically isolated signal 1 (CD3ζ) from costimulatory signal 2 (CD28) and assembled these signals into separate CARs individually targeting mesothelin and a-folate receptor (FRa). Signal 1 of the MSLN-targeted CAR molecule consisted of CD3ζ (MSLN-targeted scFv-CD3ζ), while signal 2 of the FRa-targeted CAR molecule contained the intracellular costimulatory domain of the CD28 (FRa-targeted scFv-CD28). An “AND” logic gate was applied for dual-signaling CAR-T cells to achieve highly selective antitumor efficacy.

Low cytokine secretion levels were observed when dual-signaling CAR-T cells encountered cells expressing only a single TAA, whereas enhanced cytokine secretion was exhibited when cells co-expressing both antigens were targeted in vivo. Dual CAR-T cells showed antitumor activity and long-term persistence that were greater than first-generation CAR-T cells but was equal to second-generation CAR-T cells. Notably, the traditional second-generation CAR-T cells exhibited strong cytolytic effects toward tumor as well as toward normal cells expressing the only mesothelin, partially causing serious adverse reactions, whereas dual-signaling CAR-T cells showed selective specificity for tumor cells. Hence, this “AND” logic-engineered strategy allows dual-signaling CAR-T cells to exhibit the natural biological properties of T cells, such as optimized proliferation, cytokine secretion, cytotoxicity, tumor-specific homing and “off-tumor” toxicity reduction [46] (Fig. 2a).

Recently, the “AND” logic-gated strategy has been advanced in an intelligent and customizable manner by employing a synthetic Notch receptor system, which regulates the expression of custom CAR in a transcriptional manner. Upon the binding of the first CAR (CAR1) to its cognate ligand, the SynNotch receptor is induced to release transcriptional regulators and to act in the nucleus to regulate the transcription and expression of the second CAR (CAR2). In the presence of the specific antigen targeted by CAR1, CAR2 is conditionally expressed; thus, these T cells are safely engineered to achieve the highly specific recognition and killing of tumor cells [47] (Fig. 2b).

#### Trivalent CAR T cells using the “AND” logic gate

In general, the tumor microenvironment (TME) has strong immunosuppressive properties, including physical and immune barriers, which severely impede the antitumor response of CAR-T cells. Therefore, Sukumaran et al. generated a new type of trivalent CAR-T cell with an “AND” logic gate to overcome the immunosuppression of TME in a pancreatic cancer model. Rather than adding additional CARs targeting TAAs, the novel CAR-T cells respond to only tumor-specific expression patterns. The transforming growth factor (TGFβ) and interleukin 4 (IL4), as widely accepted T cell immunosuppressive cytokines in TME, are potential targets. Hence, engineered T cell was redirected to recognize the tumor-specific antigen prostate stem cell antigen (PSCA) and immunosuppressive cytokines including TGFβ and IL4, thereby transmitting independent signals, which included antigen recognition, costimulation, and cytokine secretion. The synchronous combination of these three signals ultimately initiated T cell activation, amplification, and persistence, achieving safe, and selective cytolysis at tumor sites [48] (Fig. 2c).

#### Dual CAR-T cells using the “NOT” logic gate

The current mainstream approaches for eliminating the side effects of CAR-T cells are non-specific immunosuppression or lymphodepletion, such as high-dose corticosteroids, which are inhibitory or cytotoxicity to T cells, causing secondary complications and reducing the therapeutic efficacy [49].

In the field of adoptive cell therapy safety, the “NOT” logic gate is an effective complementary strategy to the “AND” logic gate. In a proof-of-concept study, Sadelain et al. designed an inhibitory CAR-T cell with distinct CAR molecules. One CAR contained a CD28/CD3ζ signaling domain, which activated T cells to recognize the cognate antigen (CD19-targeted scFv-CD28/CD3ζ); however, another CAR specific for prostate-specific membrane antigen (PSMA) consisted of an inhibitory signaling domain derived from PD-1 or CTLA-4 instead of a costimulatory domain (PSMA-targeted scFv-PD1/CTLA-4). The expression of PSMA is mainly in metastatic prostate cancer but is also present in normal and benign tissues [50]. iCAR-T cells selectively kill tumor cells expressing CD19 molecules, whereas off-target cells co-expressing the CD19 and PSMA recognized by iCARs were protected from attack, allowing T cells to distinguish target cells from off-target cells. It is worth noting that iCAR-mediated inhibition is transient and reversible, similar to the inhibition mediated by natural killer cells. Despite previous exposure to inhibitory antigens, iCAR-T cells maintain reactivity against targeted antigens after detaching from inhibitory antigens. Several crucial factors profoundly influence the function of iCAR-T cells, and special attention will be required in the future clinical application phase, including toward the affinity and expression level of iCARs, appropriate target antigen selection and the optimization of the CAR/iCAR ratio [51] (Fig. 2d).

## Conclusions

The clinical application of CAR T cell therapy has been limited by disease relapse caused by antigen escape and on-target, off-tumor toxicities due to low antigen specificity. To broaden the antigen coverage, T cells have been modified with multi-antigen targeting and combined with the “OR” logic gate to reduce antigen escape. Pooled, dual, tandem, and trivalent CAR-T cells with broader antigen coverage have been developed in preclinical and clinical trials. Because of the reduced antigen specificity, CAR-T cells with an “OR” logic gate could potentially have more severe on-target, off-tumor toxicities while reducing antigen escape. The “AND” and “NOT” logic gates better balance the relationship between antitumor efficacy and on-target, off-tumor toxicities. The SynNotch receptor system has applied the “AND” logic gate in an intelligent and customizable manner. In addition to adding additional chimeric antigen receptors, the “AND” logic gate can also provide engineered T cells with the ability to selectively respond to tumor-specific expression patterns. The antitumor efficiency of trivalent CAR T cells targeting PSCA, TGFβ, and IL4 has been verified in a preclinical trial. The “NOT” logic gate enhanced the safety of CAR-T cells. Future multi-targeted antigen strategies require more optimized designs and more rational logic gates to expand the CAR-T cell therapeutic window.

## Data Availability

All data generated or analyzed in this study are included in this article. Other data that are relevant to this article are available from the corresponding author upon reasonable request.

## Abbreviations

FDA: The US Food and Drug Administration
CAR-T: Chimeric antigen receptor-T
B-ALL: B cell acute lymphocytic leukemia
PD-1: Programmed death-1
CTLA-4: Cytotoxic T lymphocyte-associated protein-4
scFv: Single-chain variable fragment
ACT: Adoptive T cell therapy
CAIX: Carbonic anhydrase IX
CEA: Carcinoembryonic antigen
MSLN: Mesothelin
IL13Rα2: Interleukin 13 receptor, alpha2
FAP: Fibroblast activation protein-α
ZFN: Zinc-finger nuclease
TCR: T cell receptor
HER2: Human epidermal growth factor receptor-2
CAA: Cholangiocarcinoma
EGFR: Epidermal growth factor receptor
PR: Partial remission
PET-CT: Positron emission tomography-computed tomography
GBM: Glioblastoma
ZAP-70: Zeta-chain-associated protein kinase 70
VH: Variable heavy
VL: Variable light
R/R: Relapsed and refractory
HSCT: Hematopoietic stem cell transplantation
MRD: Minimal residual disease
CR: Complete remission
GVHD: Graft versus host disease
HLA: Histocompatibility antigen
TAA: Tumor-associated antigen
FRa: A-folate receptor
PSCA: Prostate stem cell antigen
PSMA: Prostate-specific membrane antigen
TGFβ: Transforming growth factor
IL4: Interleukin 4
GFP: Green fluorescent protein
